# An exploration of transactional states and cessation-related social variables within adolescent smokers

**DOI:** 10.1186/1617-9625-10-20

**Published:** 2012-12-20

**Authors:** Jay T Lee, Dennis W Smith, Brian Colwell

**Affiliations:** 1 Department of Educational Psychology, Health Program Area, College of Education, University of Houston, Houston, TX, USA; 2 Department of Social and Behavioral Health, Texas A&M School of Rural Public Health, College Station, TX, USA

## Abstract

**Background:**

Given the high rate of adolescent smoking, cessation remains a vital public health priority. This study explored archival data using a structured phenomenological framework known as Reversal Theory (RT). In order to better understand aspects of adolescent tobacco use we compared the transactional, psychological states described by RT to the factor structure of adolescents’ self-reported social environment influencing tobacco use.

**Methods:**

In a two step analysis of questions about self-reported tobacco use cognitions, attitudes, and behaviors from youth enrolled during the 2003–2004 period in a Texas, state-wide, mandated tobacco cessation program (N=1807), four factors and 11 items were identified as significantly related to the influence of social context and adolescents’ tobacco use. These first step results guided the items to be selected for further analysis. In step two the variables were subjected to a factor analysis using principal components extraction and varimax rotation. The resulting factor structure was compared and interpreted within the context of descriptions of RT transactional states.

**Results:**

The analysis indicated that four factors were closely aligned to descriptions of the Reversal Theory transactional states and could be reinterpreted from within the framework of RT. The first factor included feelings of self-efficacy for quitting (autic mastery). The second and third transactional factors diverged between one factor to quit, and an opposing transactional factor to continue to smoke. Both of these transactional states are variants of the autocentric state where one wants to experience feelings of gain with the help of others. The fourth factor could be interpreted as ’confidence’ or ‘optimism’.

**Conclusions:**

This intra-individual conflict revealed by the opposition of factors two and three clarifies a paradoxical issue where an adolescent wants to quit smoking with social support in one setting yet in another social environment chooses to smoke to gain or retain peer acceptance. These data illustrate that adolescent’ self-identified quit skills and social support structures are important to the quitting process. This exploratory investigation has important implications for addressing RT state reversals in youth cessation programming activities.

## Background

Rates of adolescent smoking are disconcertingly high and understanding the motivations for use – and assisting youth with quitting – has become a national focus. While most adolescents who experiment with tobacco quit before developing nicotine dependence and becoming regular smokers, a substantial minority continue to that point. While smoking rates have generally trended downward over the past decade, the trend of declines in daily youth smoking appears to have stalled, remaining at 15.6% [1].

Adolescents who regularly smoke consistently report they want to quit using tobacco and intend to attempt quitting in the future. Nationally, 54.6% of high school students say they want to quit smoking [2]. In fact, data indicate more than half of adolescent smokers have made quit attempts in the last year [2-4]. Repeated quit attempts are also quite common [5,6]. Yet many of the features influencing successful quit attempts among adolescents remain unclear. While attempts have been made to develop recommendations for assisting adolescent smoking cessation [7], these recommendations are general and insufficient, lacking ample empirical evidence to make definitive recommendations for youth cessation efforts [7-9].

### Motivations for smoking

The trajectory of an adolescent’s smoking behavior is generally predictable. Smoking attitudes, developed through observational learning and social modelling, are formed with regard to the value of tobacco use in one’s life. These attitudes predispose the young person to experimentation, and, with satisfactory experiences, escalate to regular use, eventually leading to dependence [10]. Flay, Phil, Hu & Richardson [11] have noted that, while some dispute the speed of this transition [12], the general pattern holds true.

The formation of attitudes and expectations regarding smoking are the initial step in the process, and are influenced by a variety of sources. *External factors* affecting these attitudes and expectations include implicit and explicit messages regarding social norms from peers, advertising, parental, peer and sibling modelling, etc. [13]. *Internal factors* include motivation to comply with salient others’ attitudes [14], physical and behavioral responses to initial experiences with smoking and nicotine (influenced by epigenetics and individual physiology), and the psychosocial conditioning surrounding the ceremonial act of smoking. How the individual interprets these external and internal factors is highly influenced by his or her motivational state [15].

Reversal Theory (RT) is an approach that is increasingly applied to understanding smoking behavior [15-18]. It is a multidimensional, multi-level psychological theory that is largely based on subjective experiences and meanings [15,19]. It specifically accounts for individual differences in motivations for smoking as well as moment-to-moment changes in motivational levels and states. RT has been described as a ‘grand’ meta-theory that encompasses a variety of other approaches and provides a conceptual framework for interpreting results by an examination of an individual’s experiences, as well as results from group level research. For example, where self-efficacy has been shown to be an important factor in understanding behavior, RT can be used to understand self-efficacy in relation to other motives such as sensation–seeking, rebelliousness, and the need for acceptance [15,20,21]. Thus the purpose of this study is to apply an RT approach to understanding some of the complex social variables related to quitting tobacco as self-reported by adolescents.

Seeking pleasant feelings or relief from negative affective states is frequently a reason for smoking [22]. As Waters and Sayette [23] have noted, the memories of pleasurable experiences from and during smoking may trigger relapse. Phenomenological meaning ascribed to any experience is, in a sense, processed through the complex structure of an array of metamotivational states as well as the current affective state. Actions are then influenced by these interactions. As a result of complex interactions among youths’ metamotivational states surrounding tobacco use, pleasant or unpleasant emotions accompany their use and a resulting felt transactional gain or loss. The intra-individual conflicts possible in RT states address issues important for tobacco cessation research and treatment.

In summary, little is known about the interactions among people and environment related to adolescent smokers from an RT perspective, specifically the transactional states [20]. The transactional pairs, which have not been the topic of substantial tobacco research, particularly research geared toward adolescent smokers, were the foci of the present study. More specifically, the purpose of this study was to examine the influence of transactional states upon adolescents’ self-reported smoking and cessation intentions/beliefs and the social environment influencing tobacco use.

## Methods

### Participants

This study examined archival data on 1,807 adolescents enrolled in the Texas Youth Tobacco Awareness Program (TYTAP) in 30 sites state-wide. TYTAP is a cognitive-behavioral adolescent cessation program that consists of 4, two-hour sessions conducted over the course of two weeks. The Institutional Review Boards of the University of Houston and Texas A&M University reviewed and approved the study instruments and protocol prior to the collection of data.

The subjects for this investigation came from archived data from TYTAP programs conducted during 2002 and 2003. The mean age of the 1,807 participants was 15.98 (*SD* = 1.31) years and their modal grade in school was 11^th^. Seventy percent of the sample was male, 76.9% were white and 12.0% were Hispanic. In response to a multiple-choice, categorical question, participants self-classified themselves as current smokers (61.6%), non-smokers (12.1%), occasional smokers (18.7%), or ex-smokers (7.6%). Mean cigarette consumption was 9.77 (*SD* = 8.11) cigarettes per day (CPD). Overall these distributions were representative of the data collected on youth enrolled in the program from 1998–2001. Since possession of tobacco in Texas by a minor is illegal, over 97% of the youth were referred into the program by a local court.

### Instrumentation

The investigators developed the instrument, embedded in the TYTAP first session pretest, for use in the program. It was comprised of three sections: (a) demographic variables (age, sex, and ethnicity), (b) tobacco use behaviors, and (c) tobacco use cognitive/affective variables. Tobacco use behaviors included four areas of interest: *Tobacco Use History*, including behaviors related to spit tobacco, cigarette, and cigar use (6 items), *Nicotine Dependence* (6 items), *Quitting Intentions* (3 items), and *Past Quit Attempts* (2 items). Since the tobacco use history, quitting intentions, and past quit attempts variables were continuous in nature, a fill-in-the-blank format was used for data collection. A rank order, multiple-choice format was used for the nicotine dependence variables. Tobacco use cognitive/affective variables included four areas: *Knowledge about Tobacco* (15 items, true/false format), *Attitude about Quitting* (6 items, Likert 4-scale), *Tobacco Use Peer Network* (11 items, Likert 5-scale), and *Tobacco Use Cessation Self-Efficacy* (2 items in Likert 5-scale).

The questionnaire used scales from previously validated instruments [6,24], as well as tobacco use items developed for the TYTAP program and standard demographic items. A project advisory panel of health professionals examined the content validity of the questionnaire with respect to item relevance, representativeness, and adequacy. After minor editorial improvements were completed, particularly in the wording of items, the panel judged that the questionnaire had acceptable content characteristics for use in the program [5].

### Procedures

Data from youth remanded by the court into TYTAP were analyzed in this study. Informed parental consent and informed individual assent were obtained from all participants providing data for TYTAP. Questionnaire items were part of the pretest for the cognitive/ behavioral cessation intervention. Trained TYTAP facilitators at the thirty participating sites administered the pretest to the participants during the first hour (day 1) of the four, two-hour program sessions (eight hours and four days total).

### Analysis

Procedures from the Statistical Package for the Social Sciences [25] were followed to conduct the data analyses, which proceeded in two steps. In step 1, data were subjected to an exploratory factor analysis. Based on an examination of the resulting eigenvalues, eleven variables were retained from step 1 and subjected to further analysis in step 2.

#### Step 1 analysis

The 51, pretest variables on the intake instrument were subjected to a principal component extraction and varimax rotation in order to maximize orthogonality and improve interpretability among the extracted factors. Bartlett’s Test of Sphericity produced a large approximate χ^2^ of 28703.0 (1, (n = 1176), p < .001). Where the test statistic for sphericity is large and the significance level is small, the hypothesis that the correlation matrix of these 51 variables is an identity may be rejected [26]. Kaiser-Meyer-Olkin (KMO) sampling adequacy equalled 0.886, a value close to 1. Both the KMO value and the results of Bartlett’s Test of Sphericity support the use of factor analysis.

The resulting factor structure produced thirteen factors with eigenvalues greater than 1.0 linearly aligned with 70.3% of the total variance. Four of the factors had eigenvalues greater than or equal to 2.5. Based on a standard using Kaiser’s α, factors exhibiting eigenvalues greater than 2.5 have a stronger probability of being replicated in subsequent samples [27]. The four factors could be labelled as the following latent constructs: quitting (eigenvalue (λ) = 3.8), autic mastery (λ = 3.5), autic sympathy to smoke (λ = 2.6), and autic sympathy to quit (λ = 2.5). The pattern and structure matrices were examined and eleven individual items whose coefficients exhibited the greatest linear alignment with those four factors were identified and retained for subsequent analysis.

#### Step 2 analysis

The eleven variables retained from the first analysis were subjected to a second, factor analysis using the same principal component extraction and varimax rotation method as used in step 1. The Kaiser-Meyer-Olkin sampling adequacy was 0.699, a reduction from 0.866; while this is lower than the original value it is still relatively large and considered as acceptable. Bartlett’s Test of Sphericity produced an approximate χ^2^ of 5679.19 (1, (n = 55), p < .001), meaning the hypothesis that the correlation matrix was an identity matrix is not tenable and can be rejected. No attempt was made to force a specific number of factors.

## Results

The analysis conducted in the second step recovered the original four factors, though the items and factors were reordered and aligned differently. The four factors accounted for 69.1% of the total variance instead of 70.34%, approximately the same amount from Step 1. This is an effective reduction from 51 to 11 items. Table 1 presents a list of the retained items, along with their scales, means, and standard deviations. Table 2 presents the items in the four factors and items verified in Step 2. For clarity, structure coefficients less than .30 were suppressed and are not presented in the table. 

**Table 1:** The 11 retained items, scaling, means and standard deviations^†^

**Item**	**Scale**	**Mean(SD)**
I can quit using tobacco any time I want.	Scale (1–4);	
	1=strongly agree	2.43(.901)
	4=strongly disagree	
I have the skills necessary to quit smoking/dipping.	Scale (1–4);	
	1=strongly agree	2.07(.762)
	4=strongly disagree	
Quitting smoking/dipping would be easy.	Scale (1–4);	
	1=strongly agree	2.81(.952)
	4=strongly disagree	
How confident are you that you can quit smoking totally and for good if and when you wanted to?	Scale (1–4);	
	1=very confident;	2.00(.870)
	4=not at all confident	
___of my 4 best friends would like me to quit smoking/dipping.	Scale (0–4);	
	0= none	1.42(1.548)
	4=all 4	
___of my 4 best friends don’t like my smoking/dipping.	Scale (0–4);	
	0= none	.93(1.275)
	4=all 4	
___of my 4 best friends would help me trying to quit smoking/dipping.	Scale (0–4);	
	0= none	2.26(1.661)
	4=all 4	
Smoking/dipping helps me be accepted.	Scale (1–4);	
	1=strongly agree	3.28(.664)
	4=strongly disagree	
Smoking/dipping helps me make and keep friends.	Scale (1–4);	
	1=strongly agree	3.30(.645)
	4=strongly disagree	
Do you think that you will quit smoking/dipping in the next six months?	Nominal (1–3);	
	1=yes	.70(.458)
	2=no	
	3=don’t use	
I believe that I can quit smoking/dipping if I try.	Scale (1–4);	
	1=strongly agree	1.88(.732)
	4=strongly disagree	

^**†**^A list of all 51 variables and Alpha reliabilities are available from the lead author.

**Table 2:** Step 2 Factor analysis results: means, standard deviations, and structure coefficients for adolescents Self-reported motivations to Smoke (n=1,807)

			**Structure coefficients**			
**Item**	**m**	**sd**	**1**	**2**	**3**	**4**
I can quit using tobacco any time I want.	2.41	0.896	0.869			
I have the skills necessary to quit smoking/dipping.	2.06	0.759	0.817			
Quitting smoking/dipping would be easy.	2.81	0.936	0.808			
How confident are you that you can quit smoking totally and for good if and when you wanted to?	1.99	0.867	0.780			
_____of my 4 best friends would like me to quit smoking/dipping.	1.68	1.478		0.866		
_____of my 4 best friends don’t like my smoking/dipping.	0.95	1.284		0.815		
_____of my 4 best friends would help me trying to quit smoking/dipping.	2.25	1.662		0.618		−0.345
Smoking/dipping helps me be accepted.	3.27	0.676			0.940	
Smoking/dipping helps me make and keep friends.	3.29	0.651			0.939	
Do you think that you will quit smoking/dipping in the next six months?	0.71	0.600	−0.339			0.703
I believe that I can quit smoking/dipping if I try.	1.88	0.729				0.649

As shown in Table 2, four of the eleven items aligned with Factor 1 (autic mastery or ‘self-efficacy factor’) represented the autic mastery dimension as described in RT. These items are associated with feelings of self-efficacy and confidence for quitting. One item (‘I plan to quit in the next six months’) with a structure coefficient of −0.339 was more closely aligned with Factor 4 (structure coefficient = 0.703). Three items comprise Factor 2 and identify the RT dimension for autic sympathy to quit. One of the Factor 2 items (‘Best friends would help me quit’) also loaded (negatively) on Factor 4. Factor 3 items identified autic sympathy to smoke, retaining two items that endorse the acceptance of smoking among friends. The fourth factor contained two items positively related to autic mastery (confidence or optimism) for quitting smoking, and one negative structure coefficient (‘Best friends would help me quit’).

## Discussion

The purpose of this exploratory investigation was to examine the self-reported attitudes, beliefs, and quit attempts of adolescents related to smoking and tobacco cessation within the broad context of the transactional states of RT. Applying RT constructs to self-reported data from participants in an established tobacco education and cessation program was seen as an opportunity to not only re-examine archival data but to enhance the behavioral basis of existing tobacco cessation program components targeting adolescents. The two factor analyses conducted in this study reduced 51 initial pre-test variables to 11 variables aligned with a similar amount of variance. The eleven items aligned with four factors that were interpreted within an RT transactional states framework. Factor 1 represented the variable autic mastery – feelings of self-efficacy and confidence for quitting. Factor 2 represented autic sympathy to quit and Factor 3 was autic sympathy to smoke. Like Factor 1, Factor (4) contained autic mastery-type items, but focused on the individual’s confidence and capability to quit. A cautious interpretation is that these data demonstrate that adolescents self-identify quit skills, confidence, optimism, and social support structures as important to the quitting processes. In turn, social structures surrounding adolescent users are important to continuing tobacco use when quit/continue reversals are encountered and may be perceived in paradoxical and confusing ways in differing social environments.

Previously, RT was used to investigate a number of common health behaviors that, while broad in nature, are profoundly important in understanding risky actions [28]. Apter [29] addressed the concerns of inconsistent and contradictory behaviour when he described the nature of motivation, stated that:

“…certain behaviors are engaged in because they lead to the achievement of a goal and sometimes because they are pleasurable in themselves. The differences between these two kinds of mental states are not about the goals or the means themselves, …nor are they concerned with motives as such. Rather they are about the way in which these motives are structured, interpreted, and organized within experience; they are therefore referred to as metamotivational states.” (p. 6)

This theoretical framework is useful for explaining complex behaviors and subjective experiences such as smoking. While there are a number of theories approaching the subject of motivation from a variety of perspectives, few are as comprehensive as the multidimensional application of RT.

Central to the RT approach is that individuals switch (reverse) between two stable states within identified pairs through a variety of mechanisms including satiation, frustration, and contingent events. For example, feeling high arousal at one time may be pleasurable (e.g. watching an exciting sporting event), but in the next moment the same level of arousal may be interpreted as unpleasant (e.g. one of the players becomes injured). The interpretation depends not on a single metamotivational state of the individual [30], such as arousal-seeking, but also on other metamotivational states. RT affords modeling of complex, internal interactions and apparent motivational contradictions.

**Figure 1:**
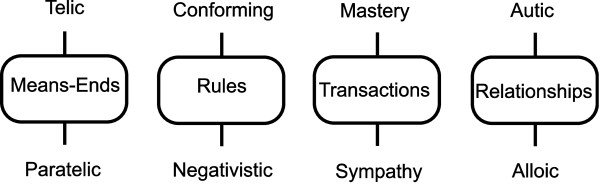
**Pairs of metamotivational states and their respective domains. **The metamotivational states of each pair are connected by whiskers to the associated domain shown in the bubble.

RT postulates four pairs of metamotivational states: telic/paratelic, conforming/negativistic, mastery/sympathy and autic/alloic, pairs. Each of the pairs is associated with a different domain as shown in Figure 1. The first of these pairs, telic/paratelic, refers to an individual’s focus on either the process or achievement (means-ends) of a goal. The next pair, the conforming/negativistic pair, is associated with one’s desire to either conform to or rebel against perceived social norms (rules). These two pairs are referred to as ‘somatic’ because the associated feelings, such as excitement, often refer to sensory experiences [31]. The somatic states are sometimes examined in combination with each other and the most frequently cited by tobacco researchers as affecting cessation, particularly as it pertains to lapses during smoking cessation [21,32]. 

The other two pairs are referred to as the transactional pairs and are crucial to understanding interactions among people and things. They include the mastery/sympathy pair and the autic/alloic pair. The mastery/sympathy pair refers to one’s interpretation of events with regard to how they affect one’s sense of power and strength. For example, in the mastery state, an individual is primarily concerned with achieving a sense of power, and “…transactions are evaluated as they bear on these issues of strength versus weakness [19], p. 67].” In the sympathy state one is more concerned with cooperation and feeling understood and accepted. The autic/alloic pair refers to whether one wants primarily to derive benefit for oneself or for another. An individual is in an autic state when he/she wants benefits for oneself. In the opposing, allocentric state one is gratified when another individual benefits.

**Figure 2:**
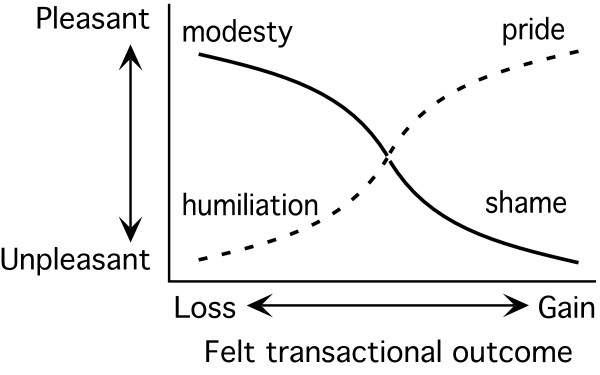
**The conceptual relationships of the autic mastery and alloic mastery state combinations **[16]**. **The autic mastery state combination is represented by the dashed line and the alloic mastery state combination by the solid line.

**Figure 3:**
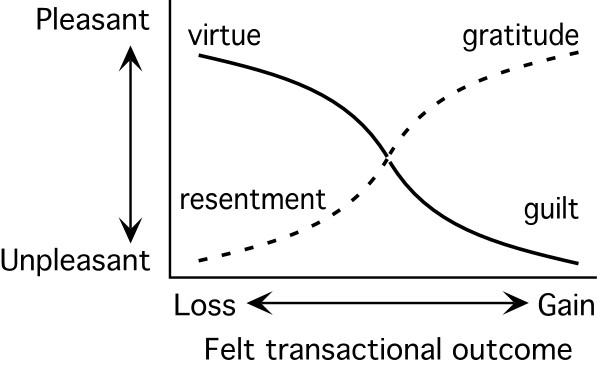
**The conceptual relationships of the autic sympathy and alloic sympathy state combinations **[16]**. **The autic sympathy state combination is represented by the dashed line and the alloic sympathy state combination by the solid line.

The dynamic combinations of the four pairs of states influence the overall, perceived pleasant and unpleasant sensations that emerge. Each state, therefore, gives rise to a *feeling* that is state-specific, and an *emotion,* which is produced in combination with other states of the somatic and transactional pairs. In this way, for example, the theoretical relationships for the autic and alloic states from the mastery and sympathy perspectives are depicted in Figures 2 and 3. As shown in Figure 2 when the autic mastery combination is the active state, an individual is likely to experience pleasant feelings when an outcome is perceived as gaining or winning. 

As previously stated, the RT transactional domain has not been studied as a part of youth tobacco cessation. However, its component parts have and are shown to be essential in behavior change. For example, autic mastery is a construct from RT that is analogous to the more familiar construct of self-efficacy, yet can be interpreted in relation to other psychological variables as a component of an inclusive theory. The pleasant emotions associated with tobacco use mastery and the sense of transactional gain creates a new framework for interpreting and extending the results of this research when other, simultaneous states are examined. The opposing combination, alloic mastery (gain by others), is associated with pleasant emotions like modesty or unpleasant emotions such as shame when personally gaining. Thus, the mastery/sympathy pair is about interactions with other people and things from the perspective of either competition or cooperation.

These points seem instructive for further testing and possible emphases in existing adolescent tobacco cessation programs. Within an RT, transactional states framework, the challenges for adolescents attempting to quit smoking include recognizing one’s transactional states and matching appropriate cognitive-behavioral quit strategies for those states. Programmers should consider the inclusion of program activities to assist participants in examining how those states interact within various social groups, social surroundings, and networks. The next four paragraphs present several, general examples of possible applications to cessation program strategies by the identified factors.

Factor 1 supports consideration of activities that increase cessation-related self-efficacy. From an RT perspective autic mastery can lend insights into internal struggles in the tobacco cessation process, possibly explaining the great inner conflicts young people face in attempting to quit, as well as staying quit, including recidivism to smoking from a temporary quit status. Building and enhancing program participants’ confidence to quit may encourage effective strategies in the cessation process. This finding parallels the results of the better practice cessation program review by McDonald et al. [33].

Peer influence is often viewed as counterproductive to healthy behavior. Yet, Factor 2 represents the belief that quitting with the support of others could result in social acceptance. Building autic sympathy activities into cessation programming underlines the positive influence of the social support in an autic sympathy state. Emphasizing the importance of non-smoking peer networks, and environmental restrictions, such as no-smoking policies, within a cessation program may potentially deter autic sympathy reversals.

Similar to Factor 2, Factor 3 represents the need for acceptance, but paradoxically to smoke or continue smoking. Whereas Factor 2 represents the belief that by quitting smoking one gains acceptance from others, Factor 3 represents the paradoxical belief that smoking helps one gain and keep friends — a more common interpretation of autic sympathy [34]. Interestingly, several opposing factors and significant cross loadings also underline the ambiguity of the Factor 3 transactional states and potential reversal triggers.

Consistent with the above, tobacco cessation activities should promote situations that lead toward reversals contrary to what smoking friends are perceived as doing. Such activities would be consistent with reportedly effective components from social-cognitive theory programs and fit well in cognitive–behavioral theory and motivational enhancement modules [35].

The remaining items aligned with the fourth factor and contained two large loadings (.70 and .64). It appears that Factor 4 is not comprised of leftover items but contains information relevant to the quitting process, particularly the conundrums that teens encounter during cessation; the two items in the factor illustrate interesting ambiguity. These items exhibited negative cross-loadings and suggest points of uncertainty and possible interference from reversals in the transactional dimension. The results provide support for including tobacco program strategies targeting confidence for quitting, recognition of transactional states, and potential reversal points.

As noted by O’Connell et al. [20], youth cessation and subsequent relapse is a patchwork of seemingly irrational behaviors, and the transactional states alternatively are responsible for feelings of pride, control, deprivation, and guilt. The structure, in particular the autic mastery variant (where all sides can pleasantly gain in social interactions) can help provide a theoretical framework to link the internal and external experiences of youth who shift frequently and yield conflicting motivations. Paradoxically, in this same state an adolescent may experience equal emotional satisfaction from attempting to quit in one social setting, and yet lapse by smoking in another social setting. This contradictory, psychological theater has been widely discussed in developmental literature [36] unrelated to tobacco cessation. For example, the personal fable for adolescent smokers might suggest, “I have the skills to quit; I can quit anytime.” Yet, one’s imaginary social audience (the sense of peers watching and judging substance use) mirrors a different perspective about tobacco use.

### Strengths and limitations

Analysis of self-reported data from the sample showed the average participant was: around 16 years of age, male, white, and a current smoker. Mean daily cigarette consumption was about half-a-pack. Almost all of the participants were referred into the program through the local courts. Given these sample characteristics, care should be taken in generalizing the findings from this exploratory study to other groups of tobacco using adolescents. Of particular note is the referral mechanism (local courts) in Texas. Also, since the design and purpose of the investigation were on the attributes of adolescent tobacco use and cessation within a framework of RT, the results need additional verification from research, perhaps using more diverse samples and measures specific to RT.

## Conclusions

Overall, the transactional states reflect the complexity and importance of internal abilities (Factor 1 and Factor 4) and external relationships and other social pressure as both motives for quitting (Factor 2) and for smoking (Factor 3). RT provides a distinctive framework to learn more about the motivational states of adolescent smokers. Examining the complexity of transactional states, motives, and paradoxes, including reversals within states, may assist with the development of increasingly effective interventions. The transactional state reversals experienced during the cessation process also demonstrate the convoluted motivations for actions and the reasons why cessation programming should not be just modified forms of prevention programming.

These results illustrate significant challenges for adolescent smokers. Adolescents have limited experience in social conflict and resolution. The developmental immaturity of adolescents in recognizing intra- and inter-personal states in social situations place them at risk for motivational states that support using tobacco. Youth tobacco-cessation program components that target recognition of transactional conflicts hold promise for delaying the use of tobacco and may facilitate cessation.

## Competing interests

The authors declare they have no significant competing financial, professional or personal interests that might have influenced the performance or presentation of the work described in the manuscript. The authors declare that they have no actual or potential competing interests.

## Authors’ contributions

The authors contributed equally to this manuscript. All authors read and approved the final manuscript.
